# The complete chloroplast genome sequence of *Taiwania flousiana*

**DOI:** 10.1080/23802359.2020.1721355

**Published:** 2020-02-03

**Authors:** Qinghua Wang, Yunqing Li, Xiaolong Yuan, Yi Wang

**Affiliations:** Laboratory of Forest Plant Cultivation and Utilization, Yunnan Academy of Forestry and The Key Laboratory of Rare and Endangered Forest Plants of State Forestry Administration, Kunming, Yunnan, People’s Republic of China

**Keywords:** *Taiwania flousiana*, chloroplast, Illumina sequencing, phylogenetic analysis

## Abstract

The first complete chloroplast genome (cpDNA) sequence of *Taiwania flousiana* was determined from Illumina HiSeq pair-end sequencing data in this study. The cpDNA is 132,565 bp in length, contains a large single copy region (LSC) of 72,930 bp and a small single copy region (SSC) of 59,477 bp, which were separated by a pair of inverted repeats (IR) regions of 79 bp. The genome contains 121 genes, including 83 protein-coding genes, 4 ribosomal RNA genes, and 34 transfer RNA genes. Further phylogenomic analysis showed that *T. flousiana* and *Taiwania cryptomerioides* clustered in a clade in Cupressaceae family.

*Taiwania flousiana* is the species of the family Cupressaceae. It is mainly distributed in China and northern Burma (Xiang et al. 2004). It is rare and precious tree species in the world and unique living fossil plants in China (Yin et al. 2018). *T. flousiana* is an excellent precious timber tree species, the wood can be used in building, making furniture and coffins, bridge and boat construction, and paper manufacture, it’s also an excellent garden greening tree species (Wei et al. 2018). *Taiwania flousiana* has great scientific value for the study of paleogeography, paleoclimate, and palaeoflora (Lian 2019). However, there has been no genomic studies on *T. flousiana.*

Herein, we reported and characterized the complete *T. flousiana* plastid genome. The GenBank accession number is MN897726. One *T. flousiana* individual (specimen number: 201903072) was collected from Kunming arboretum, Yunnan Academy of Forestry, Yunnan Province of China (25°14′20″ N, 102°75′17″ E). The specimen is stored at Yunnan Academy of Forestry Herbarium, Kunming, China and the accession number is WQH001. DNA was extracted from its fresh leaves using DNA Plantzol Reagent (Invitrogen, Carlsbad, CA, USA).

Paired-end reads were sequenced by using Illumina HiSeq system (Illumina, San Diego, CA). In total, about 23.8 million high-quality clean reads were generated with adaptors trimmed. Aligning, assembly, and annotation were conducted by CLC de novo assembler (CLC Bio, Aarhus, Denmark), BLAST, GeSeq (Tillich et al. 2017), and GENEIOUS v 11.0.5 (Biomatters Ltd, Auckland, New Zealand). To confirm the phylogenetic position of *T. flousiana*, other eight species of *Cupressaceae* family from NCBI were aligned using MAFFT v.7 (Katoh and Standley 2013). The Auto algorithm in the MAFFT alignment software was used to align the eleven complete genome sequences and the G-INS-i algorithm was used to align the partial complex sequences. The maximum likelihood (ML) bootstrap analysis was conducted using RAxML (Stamatakis 2006); bootstrap probability values were calculated from 1000 replicates. *Torreya fargesii* (KT027377) and *Torreya parvifolia* (MH744490) were served as the out-group.

The complete *T. flousiana* plastid genome is a circular DNA molecule with the length of 132,565 bp, contains a large single-copy region (LSC) of 72,930 bp and a small single-copy region (SSC) of 59,477 bp, which were separated by a pair of inverted repeats (IR) regions of 79 bp. The GC content of the LSC and SSC regions are 35.7 and 33.3%, respectively. The plastid genome contained 121 genes, including 83 protein-coding genes, 4 ribosomal RNA genes, and 34 transfer RNA genes. Phylogenetic analysis showed that *T. flousiana* and *Taiwania cryptomerioides* clustered in a unique clade in *Cupressaceae* family (Figure 1). The determination of the complete plastid genome sequences provided new molecular data to illuminate the *Cupressaceae* family evolution.

**Figure 1.**
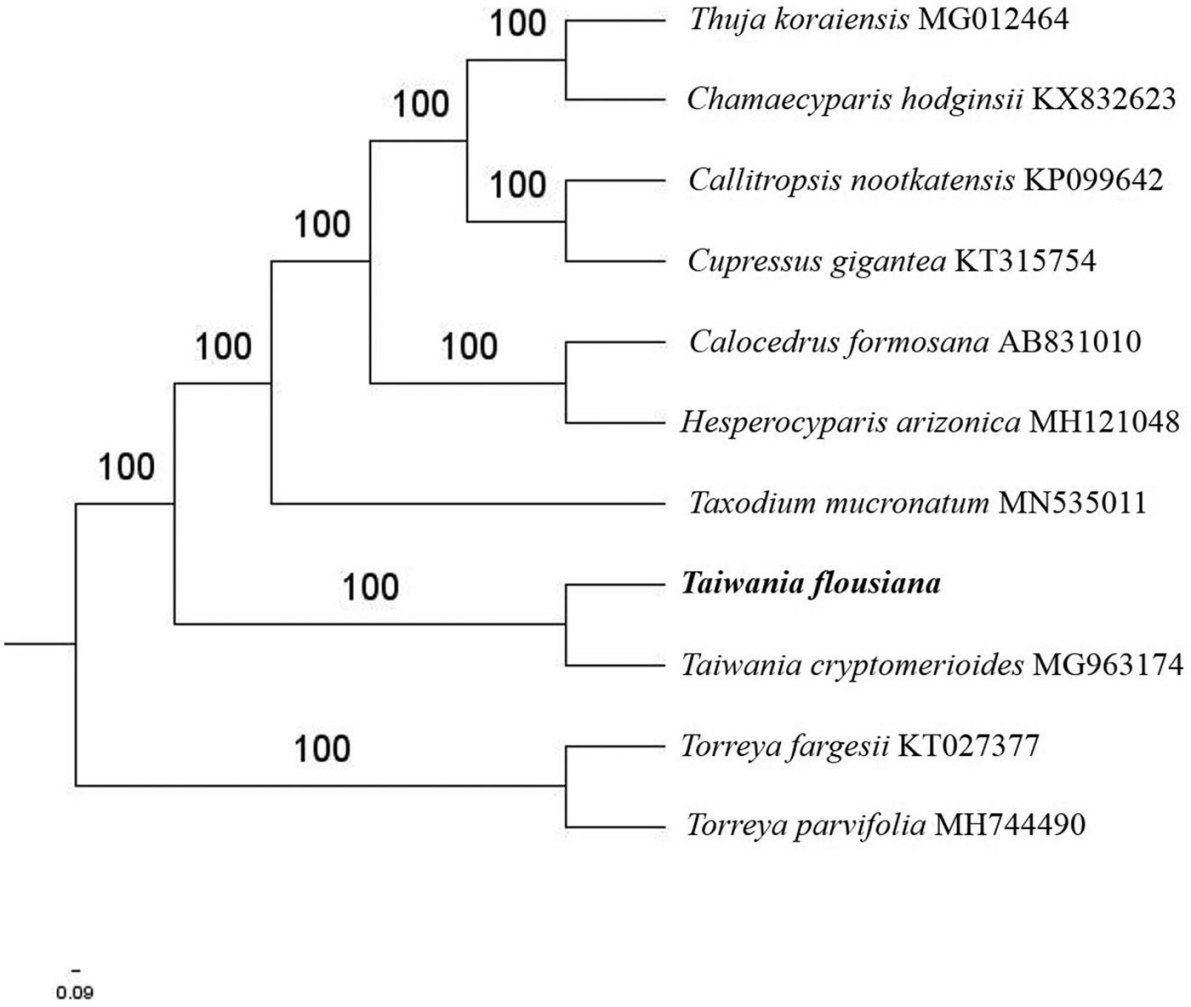
The maximum-likelihood tree based on the nine chloroplast genomes of *Cupressaceae* family. The bootstrap value based on 1000 replicates is shown on each node.
